# The effect of advanced maternal age on perinatal outcomes in nulliparous singleton pregnancies

**DOI:** 10.1186/s12884-018-1984-x

**Published:** 2018-08-22

**Authors:** Bekir Kahveci, Rauf Melekoglu, Ismail Cuneyt Evruke, Cihan Cetin

**Affiliations:** 1 Department of Obstetrics and Gynecology, Diyarbakır Gazi Yaşargil Training and Research Hospital, 21010 Diyarbakır, Turkey; 2 Faculty of Medicine, Department of Obstetrics and Gynecology, The University of Inonu, 44280 Malatya, Turkey; 3 Faculty of Medicine, Department of Obstetrics and Gynecology, The University of Cukurova, 01330 Adana, Turkey

**Keywords:** Maternal age, Nulliparity, Preeclampsia, Pregnancy outcomes, Small for gestational age

## Abstract

**Background:**

Pregnancy at advanced maternal age has become more common in both developed and developing countries over the last decades. The association between adverse perinatal outcomes and advanced maternal age has been a matter of controversy in several studies. The objective of this study is to investigate the impact of advanced maternal age on perinatal and neonatal outcomes of nulliparous singleton pregnancies.

**Methods:**

Records of patients admitted to the Department of Obstetrics and Gynecology, University of Cukurova School of Medicine, between January 2011 and July 2015 for routine mid-trimester fetal ultrasonography were retrospectively reviewed. The control (age: 18–34 years), advanced maternal age (35–39 years), and very advanced maternal age (> 40 years) groups included 471, 399, and 87 women, respectively.

**Results:**

Gestational diabetes, gestational hypertension, and cesarean delivery rates were more common in the very advanced maternal age group, with compared with the advanced maternal age and the younger age group. There were no significant differences in regarding rates of spontaneous preterm delivery before 34 weeks of gestation, prolonged rupture of membranes, large for gestational age infants, and operative vaginal delivery rates between the groups. Also, there were no significant differences regarding in APGAR scores, the rate of low birth weight infants, and neonatal morbidity rates between the groups. However, admission to the neonatal intensive care unit requirement was more common in the two advanced maternal age groups compared with the control group.

**Conclusion:**

Advanced maternal age is a risk factor for gestational diabetes mellitus, gestational hypertension, preeclampsia, small for gestational age infants, spontaneous late preterm delivery, and cesarean section, with significant potential clinical implications.

## Background

Pregnancy at advanced maternal age has become more common in both developed and developing countries over the last decades [1]. Advanced maternal age is commonly considered to be 35 years or older, whereas very advanced maternal age is defined as older than 40 or 45 years [2, 3]. Widespread use of family planning measures, postponing pregnancy because of career goals, and advances in assisted reproductive techniques contribute to this increment [4]. Accordingly, concerns about adverse effects of advanced maternal age on perinatal outcomes have also increased steadily over the past years. The association between adverse perinatal outcomes and advanced maternal age has been a matter of controversy in several studies. While some researchers have noted an increased rate of adverse pregnancy outcomes in women older than 35 years, others have failed to find any association between advanced maternal age and adverse perinatal outcomes [5, 6]. This discordance in conclusions could be attributed to the heterogeneity of study populations, differences in the definition of pregnancy outcomes, and failure to adjust for potential confounders. Therefore, we aimed to investigate the impact of advanced maternal age on perinatal and neonatal outcomes of nulliparous singleton pregnancies in this study. We hypothesized that gestational diabetes mellitus (GDM), gestational hypertension and increased cesarean delivery rate would be associated with advanced maternal age.

## Methods

The study was approved by the ethics committee of Cukurova University Faculty of Medicine. Written consent was obtained from all participants for the use of their clinical data in analysis and reporting before mid-trimester fetal ultrasonography scan. The retrospective analyses of the prospectively collected database of patients admitted to the Department of Obstetrics and Gynecology, University of Cukurova School of Medicine, between January 1, 2011, and July 1, 2015, for routine mid-trimester fetal ultrasonography was performed. Patients who met the following criteria were enrolled: 1) maternal age of 18–45 years, 2) viable singleton pregnancy, 3) nulliparity, 4) admission at 18–23 weeks of gestation for mid-trimester fetal ultrasonography, and 5) unremarkable obstetric history. The exclusion criteria were 1) any concomitant chronic disease (diabetes, hypertension, chronic renal disease), 2) body mass index > 35 kg/m^2^, 3) a history of intrauterine insemination and in vitro fertilization/intracytoplasmic sperm injection 4) a history of uterine surgery, 5) any known Mullerian anomaly, 6) presence of any uterine and adnexal mass, 5) multiple pregnancies, 6) smoking and alcohol use, 7) and any fatal congenital anomalies or chromosomal abnormalities. Main outcome variables included, gestational hypertension, preeclampsia, ablatio placenta, placenta previa, spontaneous preterm delivery before 34 weeks of gestation, spontaneous preterm delivery between 34 and 37 weeks of gestation, prolonged rupture of membranes, delivery of a (SGA) or (LGA) fetus, post-term pregnancy, operative vaginal delivery and Cesarean delivery.

GDM was diagnosed based on a positive 75-g oral glucose tolerance test result, according to the one-step diagnostic approach between 24 and 28 weeks of gestation. The diagnosis of GDM was defined based on a single glucose concentration that met or exceeded the threshold value (fasting value, 92 mg/dl; 1-h value, 180 mg/dl; and 2-h value, 153 mg/dl). Gestational hypertension was defined as systolic blood pressure ≥ 140 mmHg and/or diastolic blood pressure ≥ 90 mmHg on at least two occasions 4 h apart, developing after 20 weeks’ gestation in a previously normotensive woman in the absence of significant proteinuria and end-organ dysfunction (thrombocytopenia, renal insufficiency, impaired liver function, pulmonary edema and cerebral or visual symptoms). fined as gestational hypertension with proteinuria of ≥300 mg in 24 h, or the new-onset end-organ dysfunction. Placental abruption was defined as the premature separation of the placenta and placenta previa was described as the insertion of the placenta close to the internal cervical os. Spontaneous preterm delivery included those with spontaneous onset of labor before 37 completed weeks of pregnancy. Prolonged rupture of membranes was defined rupture of membranes occurring at least 18 h before the onset of labor. SGA and LGA were defined as those with estimated fetal weight below the 10th percentile or above the 90th percentile for gestation, respectively. Post-term pregnancy was defined as pregnancies at or beyond 42 0/7 weeks of gestation. Operative vaginal delivery was described as the accomplishment of delivery by applying direct traction on the fetal skull with forceps or by applying traction to the fetal scalp using a vacuum extractor. Cesarean delivery was defined as a surgical procedure to use the delivery of a fetus by abdominal route. The neonatal intensive care unit (NICU) admission criteria were defined as follow; low birthweight (< 2500 g), prematurity (36 weeks gestation or less), respiratory problems (apnea or cyanotic episodes, any respiratory distress causing concern), suspicion of infection with clinical concern, gastrointestinal problems (feeding problems, bile stained vomiting, or other clinical signs suggesting bowel obstruction), metabolic problems, central nervous system problems (convulsion, neonatal encephalopathy), cardiovascular problems requiring monitoring or intervention and any baby that is causing concern to the attending doctor that the baby requires observation or treatment in NICU.

A total of 957 women met the study criteria, of which 471 were assigned to the control group (aged 18–34 years) whereas 399 and 87, respectively, formed the advanced maternal age (35–39 years) and very advanced maternal age (40 years or more) groups. Antenatal care and delivery for patients included in this study took place at our clinic, according to the standard protocols. Maternal characteristics, perinatal and neonatal outcomes were obtained from medical records.

The statistical software package IBM SPSS, version 22.0 (SPSS IBM, Armonk, NY, USA), was used for all data analyses. Thus, adjusted odds ratios and 95% confidence intervals were calculated for maternal age and potential confounding factors in relation to pregnancy outcome. Differences were considered significant when *p*-values were < 0.05.

## Results

During the study period, 2617 singleton deliveries occurred at the University of Cukurova School of Medicine Department of Obstetrics and Gynecology. Of these, 957 met the study criteria. The demographic characteristics of the study population are summarized in Table 1. There were no significant differences between the groups regarding age, gravidity, the degree of consanguinity with the spouse, and educational status. In contrast, invasive prenatal diagnostic procedures were performed more often in patients aged 40 years or older.

**Table 1:** Patient characteristics

	Maternal Age Groups			*p*		
Maternal characteristics	< 35 years (a) (*n* = 471)	35–39 years (b) (*n* = 399)	≥40 years (c) (*n* = 87)	a vs. b	a vs. c	b vs. c
Age (year)^a^	27.6 ± 4.2	36.6 ± 1.4	41.71 ± 2.0	< 0.001	< 0.001	0.035
Gravidity^b^	1.0 (1.0–2.0)	1.0 (1.0–3.0)	1.0 (0.0–3.0)	0.865	0.759	0.980
Body Mass Index (kg/m^2^)^a^	28.7 ± 4.1	29.9 ± 5.8	30.1 ± 4.6	0.450	0.375	0.681
Consanguinity between spouses (first or second degree)^c^	26 (5.5)	19 (4.7)	3 (3.4)	0.284	0.120	0.330
Underwent invasive prenatal diagnosis procedure^c^	24 (5.1)	26 (6.5)	23 (26.4)	0.530	< 0.001	< 0.001
Educational status^c^				0.101	0.085	0.253
Primary school	12 (2.5)	19 (4.7)	6 (6.9)			
High school	81 (17.2)	76 (19.1)	17 (19.5)			
Graduated or higher	378 (80.3)	304 (76.2)	77 (88.5)			

^a^Data are given as mean ± SD

^b^Data are given as median (minimum-maximum)

^c^Data are presented as n (%)

Values with the same superscript letter within a row do not differ significantly at the 0.05 level

Gestational hypertension, GDM, and cesarean delivery rates were higher in the very advanced maternal age group than in the advanced maternal age and control groups. While the frequencies of preeclampsia and SGA were similar in patients aged 35–39 years and 40 years or older, the risk of preeclampsia and SGA was significantly lower in women younger than 35 years. Post-term pregnancy was significantly more common in the < 35 years age group than in the other groups. There were no significant differences in spontaneous preterm delivery before 34 weeks of gestation, prolonged rupture of membranes, ablatio placentae, placenta previa, LGA, and operative vaginal delivery rates between the groups. The frequencies of complications according to maternal age group are described in Table 2.

**Table 2:** Frequency of perinatal outcomes according to maternal age group

		Maternal Age Groups			*p*		
Perinatal Outcomes	Total	< 35 years (a) (*n* = 471)	35–39 years (b) (*n* = 399)	≥40 years (c) (*n* = 87)	a vs.b	a vs. c	b vs. c
Gestational diabetes mellitus	101	27 (5.7)	57 (14.3)	15 (17.2)	< 0.001	< 0.001	0.041
Gestational hypertension	58	20 (4.2)	29 (7.2)	8 (9.2)	< 0.001	< 0.001	0.035
Pre-eclampsia	62	22 (4.6)	31 (7.7)	8 (9.2)	0.033	< 0.001	0.457
Ablatio placenta	7	2 (0.4)	4 (1.0)	1 (1.1)	0.073	0.051	0.865
Spontaneous preterm delivery before 34 weeks	83	39 (8.2)	36 (9.0)	7 (8.0)	0.469	0.754	0.601
Spontaneous late preterm delivery between 34 and 37 weeks of gestation	71	34 (7.2)	28 (7.0)	9 (10.3)	0.342	0.044	0.038
Prolonged rupture of membranes	41	21 (4.5)	16 (4.0)	4 (4.6)	0.780	0.913	0.612
Small for gestational age	80	21 (4.5)	48 (12.0)	11 (11.5)	< 0.001	< 0.001	0.560
Large for gestational age	21	7 (1.5)	12 (3.0)	2 (2.3)	0.072	0.134	0.411
Placenta previa	24	13 (2.8)	9 (2.2)	2 (2.3)	0.613	0.218	0.891
Post-term pregnancy	66	41 (8.7)	21 (5.3)	4 (4.6)	0.021	< 0.001	0.139
Operative vaginal delivery	16	9 (1.9)	6 (1.5)	1 (1.1)	0.451	0.112	0.207
Cesarean delivery	396	175 (37.1)	167 (41.8)	44 (50.5)	0.029	< 0.001	0.040

Data are presented as n (%)

The rates of low birth weight and first- and fifth-minute APGAR scores of neonates were similar between the groups. While admission to the neonatal intensive care unit was more frequent in the advanced and very advanced maternal age groups compared with the control group, the neonatal morbidity rates were similar among the groups. The neonatal outcomes are shown in Table 3.

**Table 3:** Frequency of neonatal outcomes according to maternal age group

		Maternal Age Groups			*p*		
Neonatal Outcomes	Total	< 35 years (a) (*n* = 471)	35–39 years (b) (*n* = 399)	≥40 years (c) (*n* = 87)	a vs.b	a vs. c	b vs. c
Low birthweight < 2500 g	110	54 (11.4)	46 (11.5)	10 (11.4)	0.980	0.999	0.987
First minute APGAR score < 7	194	91 (19.3)	83 (20.8)	18 (20.7)	0.793	0.788	0.960
Fifth minute APGAR score < 7	42	22 (4.6)	17 (4.2)	3 (3.4)	0.810	0.209	0.311
Neonatal intensive care unit requirement	185	77 (16.3)	89 (22.3)	17 (19.5)	0.019	0.041	0.027
Neonatal morbidity	66	32 (6.8)	27 (6.7)	6 (6.8)	0.955	0.989	0.881

Data are presented as n (%)

Neonatal morbidity includes; sepsis, pneumonia, respiratory distress syndrome, bronchopulmonary dysplasia, intraventricular hemorrhage, necrotizing enterocolitis

According to the results of unadjusted logistic regression analysis, the rates of perinatal outcomes such as gestational diabetes, gestational hypertension, spontaneous preterm delivery before 34 weeks of gestation spontaneous late preterm delivery between 34 and 37 weeks of gestation, SGA, and cesarean delivery increased with maternal age, with the most substantial increases seen in gestational diabetes and cesarean delivery rates in the very advanced age group (OR 2.41, 95% CI 2.13–3.76; OR 2.67, 95% CI 1.90–3.82, respectively). However, there was little evidence of increased risk of ablatio placentae, prolonged rupture of membranes, LGA, and operative vaginal delivery. Babies born to older women were at higher odds of neonatal intensive care unit admission (OR 1.54, CI 95% 1.13–2.12; OR 1.69, CI 95% 1.02–2.76 at 35–39 years old and ≥ 40 old, respectively). After adjusting for gravidity, educational status, consanguinity status, and cesarean delivery rates, all these adverse outcomes remained significantly higher in older primiparae. Crude and adjusted ORs for adverse perinatal and neonatal outcomes according to maternal age are shown in Table 4.

**Table 4:** Crude and adjusted Odds ratios (95% confidence intervals) for maternal and neonatal outcomes at advanced and very advanced maternal age groups compared with < 35 years group

	Maternal Age Groups			
Perinatal Outcomes	35–39 years		≥40 years	
	Crude OR (95% CI)^b^	Adjusted OR^a^ (95% CI)^b^	Crude OR (95% CI)^b^	Adjusted OR^a^ (95% CI)^b^
Gestational diabetes	1.15 (1.01–1.27) *p* < 0.15	1.21 (0.82–1.27) *p* < 0.01	2.41 (2.13–3.76) *p* < 0.15	2.23 (0.25–3.96) *p* < 0.01
Gestational hypertension	1.57 (1.16–2.07) *p* < 0.15	1.55 (1.32–1.78) *p* < 0.01	1.70 (1.12–2.63) *p* < 0.15	1.68 (0.69–4.16) *p* < 0.01
Pre-eclampsia	1.26 (1.15–1.39) *p* < 0.15	1.34 (1.11–1.62) *p* < 0.01	1.44 (1.21–1.67) *p* < 0.15	1.37 (1.13–1.48) *p* < 0.01
Ablatio placenta	1.00 (0.63–1.57) *p* = 0.99	1.00 (0.42–1.68) *p* = 1.00	1.00 (0.39–2.64) *p* = 1.00	1.03 (0.91–1.12) *p* = 0.88
Spontaneous preterm delivery before 34 weeks	0.86 (0.68–0.98) *p* < 0.15	0.90 (0.75–1.16) *p* = 0.03	1.35 (0.95–1.91) *p* < 0.15	1.33 (1.21–1.49) *p* = 0.24
Spontaneous late preterm delivery between 34 and 37 weeks of gestation	0.61 (0.29–1.32) *p* = 0.20	0.82 (0.72–1.08) *p* = 0.35	1.22 (1.10–1.64) *p* < 0.15	1.41 (1.29–1.57) *p* < 0.01
Prolonged rupture of Membranes	0.87 (0.85–0.91) *p* = 0.78	0.91 (0.71–1.15) *p* = 0.81	0.83 (0.72–0.95) *p* = 0.94	1.05 (0.72–1.08) *p* = 0.98
SGA (<10th centile)^b^	1.71 (1.44–2.49) *p* < 0.15	1.65 (0.71–3.05) *p* < 0.01	1.63 (1.38–2.11) *p* < 0.15	1.55 (1.41–1.88) *p* < 0.01
LGA (>90th centile)^b^	1.10 (0.93–1.32) *p* = 0.27	1.12 (0.81–1.52) *p* = 0.35	0.68 (0.34–1.37) *p* = 0.27	0.54 (0.50–0.64) *p* = 0.11
Placenta previa	1.00 (0.84–1.23) *p* = 0.95	1.05 (0.81–1.15) *p* = 0.88	0.78 (0.66–0.98) *p* = 0.15	1.03 (0.76–1.34) *p* = 0.25
Post-term pregnancy	1.05 (0.75–1.54) *p* = 0.61	1.02 (0.74–1.67) *p* = 0.77	0.68 (0.45–1.03) *p* = 0.21	0.54 (0.41–0.67) *p* = 0.18
Operative vaginal delivery	0.72 (0.46–1.18) *p* = 0.19	0.68 (0.51–0.88) *p* = 0.34	0.81 (0.72–0.96) *p* = 0.15	0.73 (0.56–0.94) *p* = 0.33
Cesarean delivery	1.75 (1.61–1.95) *p* < 0.15	1.87 (1.44–2.25) *p* < 0.01	2.67 (1.90–3.82) *p* < 0.15	2.76 (1.70–4.65) *p* < 0.01
Low birth weight < 2500 g	1.23 (1.08–1.41) *p* < 0.15	1.27 (1.14–1.59) *p* < 0.01	1.20 (0.84–1.23) *p* < 0.15	1.19 (1.09–1.35) *p* < 0.01
First minute APGAR score < 7	1.14 (0.91–1.49) *p* < 0.15	1.10 (0.73–1.56) *p* = 0.08	1.27 (0.95–1.71) *p* < 0.15	1.26 (1.01–1.55) *p* = 0.04
Fifth minute APGAR score < 7	0.94 (0.71–1.30) *p* = 0.74	1.00 (0.99–1.05) *p* = 0.96	1.04 (0.95–1.15) *p* < 0.15	1.05 (1.00–1.08) *p* = 0.04
Neonatal intensive care unit requirement	1.69 (1.02–2.76) *p* < 0.15	1.68 (1.42–2.15) *p* < 0.01	1.54 (1.13–2.12) *p* < 0.15	1.52 (1.21–1.92) *p* < 0.01
Neonatal morbidity	0.93 (0.27–3.10) *p* = 0.93	0.86 (0.70–1.09) *p* = 0.25	1.12 (0.82–1.55) *p* = 0.48	1.09 (0.90–1.33) *p* = 0.23

^a^ Adjusted for gravidity, educational status, consanguinity status and C/S rates

^b^*CI* confidence interval, *LGA* large-for-gestational age, *OR* odds ratio, *SGA* small-for-gestational age

## Discussion

Several studies investigating the impact of advanced age on pregnancy outcomes [7–9] produced conflicting results because of differences in study group homogeneity and inadequate control for variables like maternal diseases, assisted conception, obesity, multiple pregnancies, and parity. The results of this study, in which all of these variables were controlled for, demonstrate that advanced maternal age nulliparous women with no previous chronic diseases, including obesity, there is an increased odds of adverse perinatal and neonatal outcomes, including gestational diabetes, gestational hypertension, preeclampsia, SGA, spontaneous late preterm delivery between 34 and 37 weeks of gestation, and cesarean delivery, but not spontaneous preterm delivery before 34 weeks, ablatio placentae, prolonged rupture of membranes, placenta previa, LGA, and operative vaginal delivery. These evidence-based conclusions should be of interest to both advanced maternal age women and medical professionals. Consistently with the current results, Khalil et al. demonstrated that preeclampsia, SGA, GDM, and cesarean delivery were more common in advanced maternal age pregnancies. However, they found that gestational hypertension and spontaneous preterm delivery rates were similar after adjustment for maternal characteristics and obstetric history, and only an increased risk of iatrogenic early preterm delivery attributed to the increased rates of preeclampsia and SGA was observed in this age group [10]. In a retrospective cohort study of the impact of parity on adverse perinatal outcomes in advanced maternal age pregnancy conducted by Baser et al., preterm delivery rates were higher in the advanced maternal age group (OR 1.08, 95% CI 0.95–1.12) [11]. These contradictory results regarding the relationship between maternal age and the increased preterm delivery rate could be attributed to methodological differences, in particular, the definition of gestational age of preterm delivery, which ranged between 37, 34, and 32 weeks.

In this study, the risks of preeclampsia and gestational hypertension were significantly higher in pregnant women aged over 35 years compared with younger controls. In agreement, Duckitt et al. demonstrated that maternal age greater than 40 years doubles the risk of preeclampsia [12]. Furthermore, Saftlas et al. showed that the risk of preeclampsia increases by 30% per year after the age of 35 years old [13]. In contrast, Cleary-Goldman et al. did not find any association between hypertensive disorders of pregnancy and maternal age in The First and Second Trimester Evaluation of Risk trial. This was interpreted to be a consequence of controlling for covariates associated with gestational hypertension and preeclampsia, such as parity and assisted reproductive care [14].

The incidence of glucose intolerance increases with age owing to reduced insulin sensitivity and increased level of serum lipids. In this study, the risk of GDM increased with maternal age. However, we did not observe an age-related increase in the risk of LGA. Fulop et al. explained the reduction in insulin sensitivity with age by progressive deterioration of pancreatic β-cell function [15]. In a population-based study conducted by Carolan et al. to determine maternal and perinatal outcomes of pregnancies in women aged 45 years or older, the odds of gestational diabetes (OR 2.05, 95% CI 1.3–3.3) were greater in this age group, with the GDM incidence of 9.7% [16].

In this study, the rate of cesarean section increased linearly with maternal age, which is consistent with previous studies. In a systematic review of twenty-one studies, Bayrampour et al. found an increased risk of cesarean birth among women of advanced maternal age compared with younger women for both nulliparas and multiparas [17]. This increasing rate of the CS could be explained by the age-related weakening of the myometrium, reduction in the number of oxytocin receptors, the lower clinical threshold for obstetric interventions, and increased rates of maternal systemic diseases and obstetric complications. The result of this study also indicated increased cesarean rate as high as 37.1% in nulliparous young age women that may be accepted as low-risk pregnancies by considering the exclusion and inclusion criteria of the study. Consistently, a recent observational study revealed an increasing CS rate in low-risk pregnancies, and it was demonstrated that this rising trend in CS rates is most prominent in nulliparous women with a term cephalic in spontaneous labor [18]. Despite the data from large-scale studies implying that optimal CS rates are between 15 and 19% [19], current CS rates both worldwide and even in Turkey considerably higher than these recommended optimal primary CS rates. Reasons for this high prevalence of CS rates are complex and appear to demographic, sociocultural, and institutional factors.

The relationship between SGA and maternal age is believed to be U-shaped, with an increased risk in women under the age of 30 and over the age of 40 years old. We demonstrated that advanced maternal age is associated with a risk of SGA. Previously, Odibo et al. identified a positive dose-response relationship between advanced maternal age and increased risk of intrauterine growth restriction (IUGR). They noted that advanced maternal age is an independent risk factor for IUGR and suggested that screening for IUGR should be conducted among women aged 35 years or older [20]. Although the exact mechanism of the association between advanced maternal age and SGA has not been demonstrated clearly, it has been suggested that poor oxygen exchange may be the underlying factor.

In this study, we also examined the impact of advanced maternal age on neonatal outcomes. Admission to a NICU was more likely in the advanced maternal age groups (OR 1.69, 95% CI 1.02–2.76; OR 1.54, 95% CI 1.13–2.12 in the 35–39 and > 40 years old groups, respectively), but there were no significant differences in APGAR scores, occurrence of low birth weight, and neonatal morbidity rates between the groups. Mutz-Dehbalaie et al. investigated the impact of advanced maternal age on the rate of perinatal mortality in 56,517 singleton hospital deliveries between 1999 and 2008 in Austria. They found that the rate of perinatal mortality was more than two-fold greater in women older than 40 years than in younger women. However, there was no significant correlation between neonatal mortality rate and maternal age [21]. Delbaere et al. found increased rates of low birth weight (adjusted OR 1.69, 95% CI 1.47–1.94), very low birth weight (adjusted OR 1.62, 95% CI 1.15–2.28), and extremely low birth weight (adjusted OR 2.14, 95% CI 1.29–3.56) in primiparas of advanced maternal age after controlling for confounding factors [22]. The observed inconsistencies in the occurrence of low birth weight could be explained by the inability to control for congenital malformations and maternal diseases in the study group.

The main limitation of this study was that the results of this study are applicable to a narrow population of patients for the large list of exclusion criteria. No inferences can be made to women with previous births, history of diabetes, obesity and the other excluded variables. This failure to examine the different population may be addressed in future studies. Other limitations include the single center population, small number of women in the sample with age > 40 years and the lack of data on race/ethnicity, subfertility issues and the reasons of women for delayed pregnancy.

## Conclusions

This study showed a significant association between advanced maternal age and gestational diabetes, gestational hypertension, preeclampsia, increased cesarean section rates, SGA, and spontaneous late preterm delivery. These findings may have significant implications in clinical practice. Modification of follow-up protocols to account for these age-related risk factors could improve pregnancy outcomes in women of advanced maternal age.

## Abbreviations

CI: Confidence interval
IUGR: Intrauterine growth restriction
LGA: Large for gestational age
NICU: Neonatal intensive care unit
OR: Odds ratio
SGA: Small for gestational age
